# Institutional Notes and News

**Published:** 1911-08-12

**Authors:** 


### Institutional Notes and News.

**The Work of the West Ham Aid Committee.**

Last Saturday a garden fête, organised by the Ladies' Committee of the West Ham and Eastern General Hospital, was held in the grounds of Salway House, Woodford Green, by permission of Sir John Reynolds Roberts, who also provided a firework display, the announcement of which doubtless assisted in drawing the large gathering. The Ladies' Committee was greatly assisted by the Aid Committee of the hospital, an organisation of local residents who, though not officially connected with the hospital, have organised a committee for the purpose of arranging functions and fixtures for the benefit of the funds of the institution. Sir John Roberts and Mrs. Isit, his niece, entertained a party which included Sir John and Lady Bethell, Mr. Thos. Alex. Cook, chairman of the hospital, Mrs. Cook, chairman of the Ladies' Committee, Mr. Gurney Fowler, J.P., Mr. and Mrs. W. H. Brown, Dr. Nicoll, chairman of the Committee of the Honorary Medical Staff of the Hospital, the matron, Miss Sordy, and the secretary, Mr. A. W. Scrivener, besides a very large number of the committee and supporters. No entertainment proved more attractive than the performance given by a group of children from Star Lane, Canning Town, under the direction of Miss Ware. They danced and sang with the grace generally associated with more delicately nurtured children who have had an expensive training in folk-song. They had seldom, if ever, seen such gardens and they had never seen fireworks except penny squibs, so their delight knew no bounds when they were allowed to stay on. Considering that the teachers had undertaken to deliver each to its respective home, this was a great concession.

**Miller General Hospital Extension.**

Not long ago the Earl of Dartmouth, accompanied by the Countess of Dartmouth, laid the foundation-stone of the extension of the Miller General Hospital, Greenwich, which is to cost £22,000. Mr. John H. Robinson, chairman of the Board of Management, said that the building would be five storeys high, and would contain rooms for surgeons, anæsthetists, and the usual theatres. At present it was arranged to open two wards, and in time to open a children's ward, which was much needed in that part of South London. The hospital is over a hundred years old, and at present the accommodation was limited to twenty-five patients, but by a stretch they had been able to accommodate twenty-eight. The extension would provide for between forty and fifty patients. He announced a gift of £3,000 from the trustees of the Annie Zunz Fund on condition that the Board of Management would name a ward after them. The chairman added that they would be only too pleased to carry out the wishes of the bequest. Dr. Willes, senior physician, proposed a vote of thanks to the Earl and Countess of Dartmouth, which was seconded by Mr. Leonard Miller, vice-chairman of the board of management. The stone laying was preceded by a short religious service, and the Earl was presented with a silver trowel by the architect, Mr. Keith Young, who, with Mr. Henry Hall, has been entrusted with the work. The builder is Mr. William Nash. Mr. John Poland's "Records" of the Hospital, which was published by subscription in 1893, was referred to by Mr. Robinson, who recalled that the hospital's name was in memory of Canon Miller, one time Vicar of Greenwich, who was, of course, the founder of the Hospital Sunday movement. The name, he added, is not due to the contiguity of certain flour mills which grace the neighbourhood. Mr. Robinson also referred to the recent institution of a weekly ward, and to the great need for further beds, which the new "William Buckwell" wing will provide.

**Another "Royal" Hospital.**

It is understood that the Huddersfield King Edward Memorial Fund, which now amounts to £23,195, will endow additional accommodation at the infirmary, and that the King has graciously signified his pleasure that the privilege of using the prefix "Royal" in the name of the infirmary should be permitted. It will be remembered that the Bolton Infirmary, or a section of its committee, has been considering the advisability of petitioning the King for a similar honour, the advantages of which were explained in The Hospital of June 17, page 293.

**Insurance and Leicester Infirmary.**

Sir Edward Wood, J.P., presided at the half-yearly meeting of the Governors of Leicester Infirmary recently. During the half-year the average stay of in-patients was twenty-seven days. The larger accommodation had enabled patients to be retained five days longer than had been possible before. All items of income were larger than in the corresponding half-year of 1910, except legacies, which had fallen from £1,062 to £209. The necessity for modernising the Children's Hospital indicates that an additional ward as well as the extension of the balcony system is needed, together with a cold storage system. Sir Edward Wood referred to the anxiety which existed as to the future of the voluntary hospital. It seemed quite clear that there would not be the demand on the out-patients' department if the Bill were passed as drawn. He believed the Governors would not be dismayed at this statement because there was a good deal of anxiety about the value and character of out-patients' work. With regard to in-patients, he was of opinion that this branch of their work would be considerably increased. This raised the question of upkeep of the hospitals, and referring to the three means, municipalisation, State-aid, and the voluntary system, experience, he said, had favoured the voluntary system, and it would be a calamity if the hospitals were municipalised. With regard to the Saturday Hospital Fund, which last year produced £13,000, he was of opinion that they would certainly retain the working-men as contributors to the fund, but he did have some doubts about the women's payments, although he hoped that they would realise the enormous advantage to be derived from the small weekly contribution, and that whereas now they received nothing for sick pay under the Bill they would receive 7s. 6d. per week. The Report was seconded by Mr. S. Francis Stone, treasurer, and approved.

**A Monyhull Colony for Leicester.**

The problem of the feeble-minded in Leicestershire has been considered by the Leicester Board of Guardians, to which a provisional committee recommends the joint establishment by the Guardians of Leicester and Rutland, together with those of the Borough of Leicester, of an institution for epileptics and feeble-minded persons on the lines of the Birmingham, Aston, and King's Norton Joint Poor-law establishment, known as the Monyhull Colony. It is also suggested that a joint committee be appointed (subject to the approval of the Local Government Board) with power to purchase a site from 200 to 300 acres, and to incur preliminary expenses for the erection of such buildings as may be approved. The cost of the site and buildings is not to exceed a total sum of £50,000. Mr. J. L. Harrison, in submitting the report, pointed out that of the several sites offered one or two were very adaptable for the purpose. It was expected the site would cost £15,000 and the building and equipment £35,000. The report was adopted.

